# Supplementary material for: Oilseed Cakes: A Promising Source of Antioxidant, and Anti-Inflammatory Agents—Insights from Lactuca sativa
Source: Int J Mol Sci. 2024 Oct 15;25(20):11077. doi: 10.3390/ijms252011077 (PMC11507441; doi:10.3390/ijms252011077)
Supplement: Supplementary file 1 [file ijms-25-11077-s001.zip › ijms-3231861-supplementary.pdf]

Oilseed Cakes: A Promising Source of Antioxidant, and Anti-Inflammatory Agents—Insights from *Lactuca sativa*

Mayye Majed [1], Amal A. Galala [1,2], Mohamed M. Amer [1], Dirk Selmar [3,*] and Sara Abouzeid [1,3,*]

[1] Pharmacognosy Department, Faculty of Pharmacy, Mansoura University, Mansoura 35516, Egypt

[2] Pharmacognosy Department, Faculty of Pharmacy, Horus University in Egypt (HUE), New Damietta 34517, Egypt

[3] Institute for Plant Biology, TU Braunschweig, Mendelssohnsstr. 4, 38106 Braunschweig, Germany

* Correspondence: d.selmar@tu-bs.de (D.S.); sara86@mans.edu.eg (S.A.)

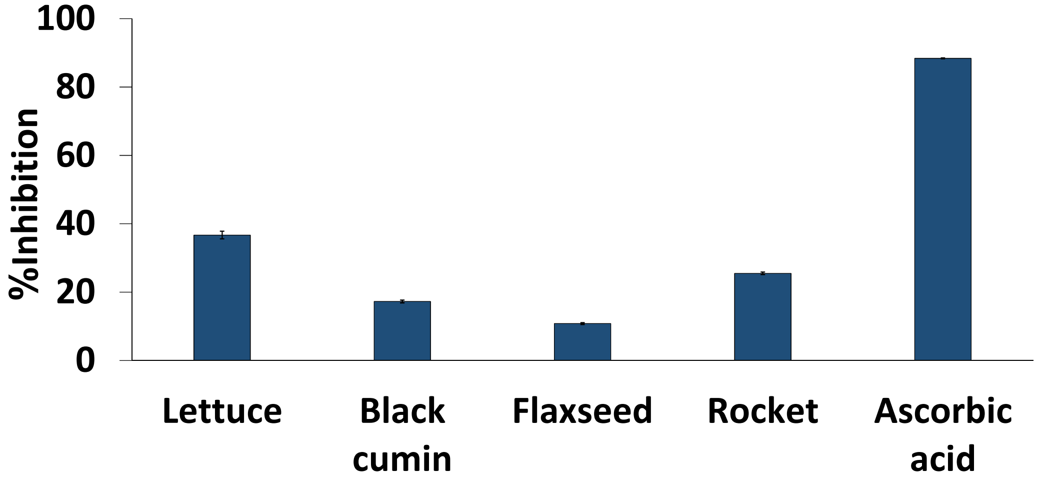

Figure S1. % Inhibition of tested seedcake methanolic total extracts in ABTS assay using ascorbic acid as standard (1mg/ml).

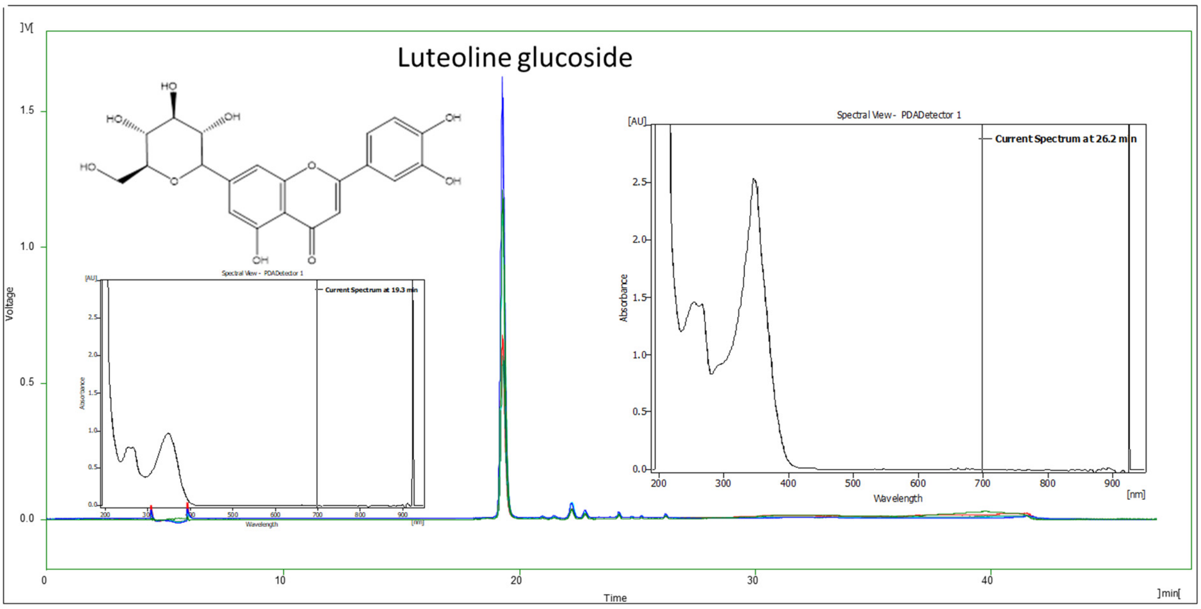

Figure S2. HPLC and UV spectrum of the luteolin glucoside standard and UV spectrum of compound at Rt 26 nm. Compounds were monitored using a photo diode array (PDA) detector at 280, 350 nm.

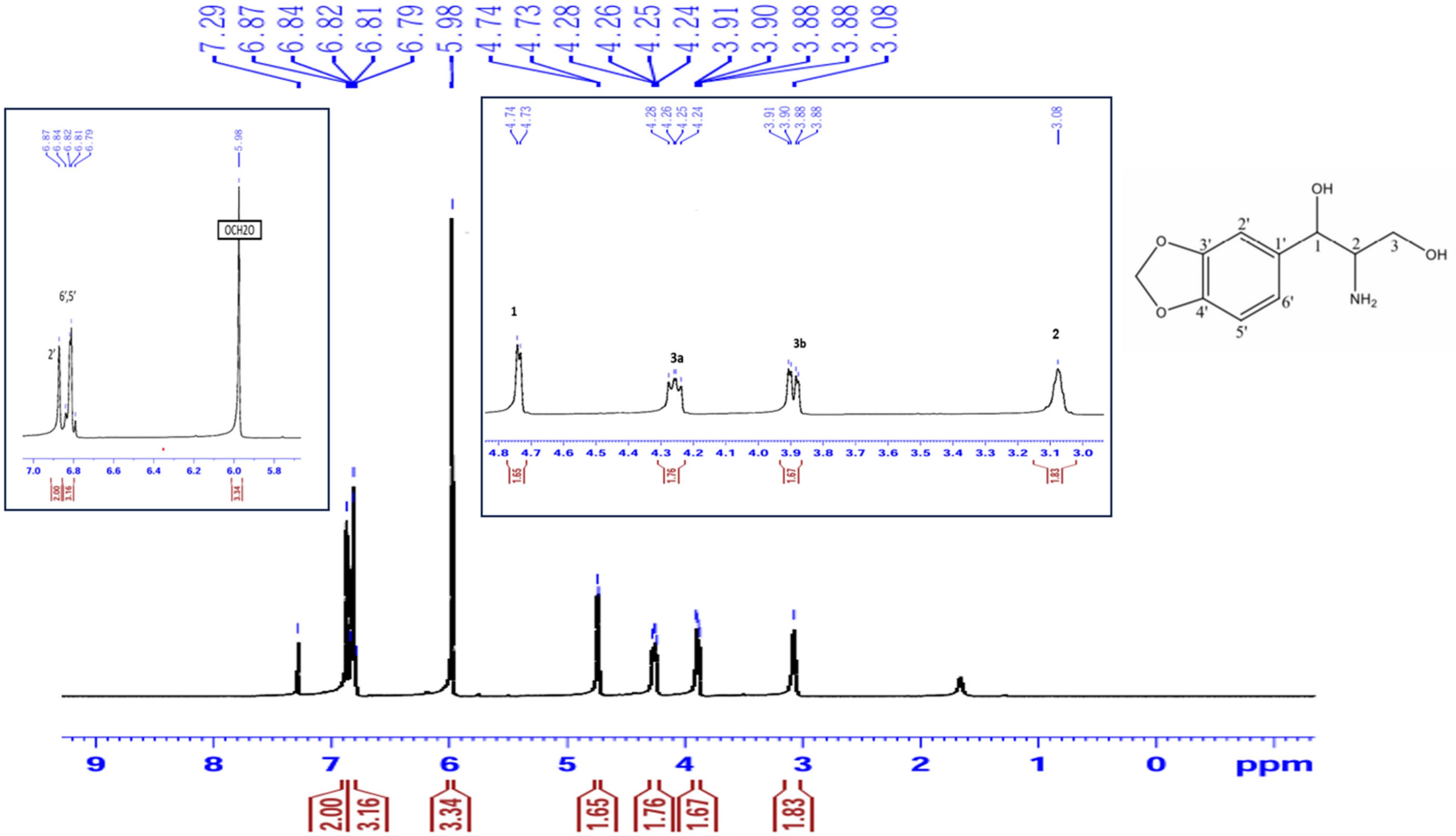

Figure S3. ^{1}H-NMR spectrum of compound 1

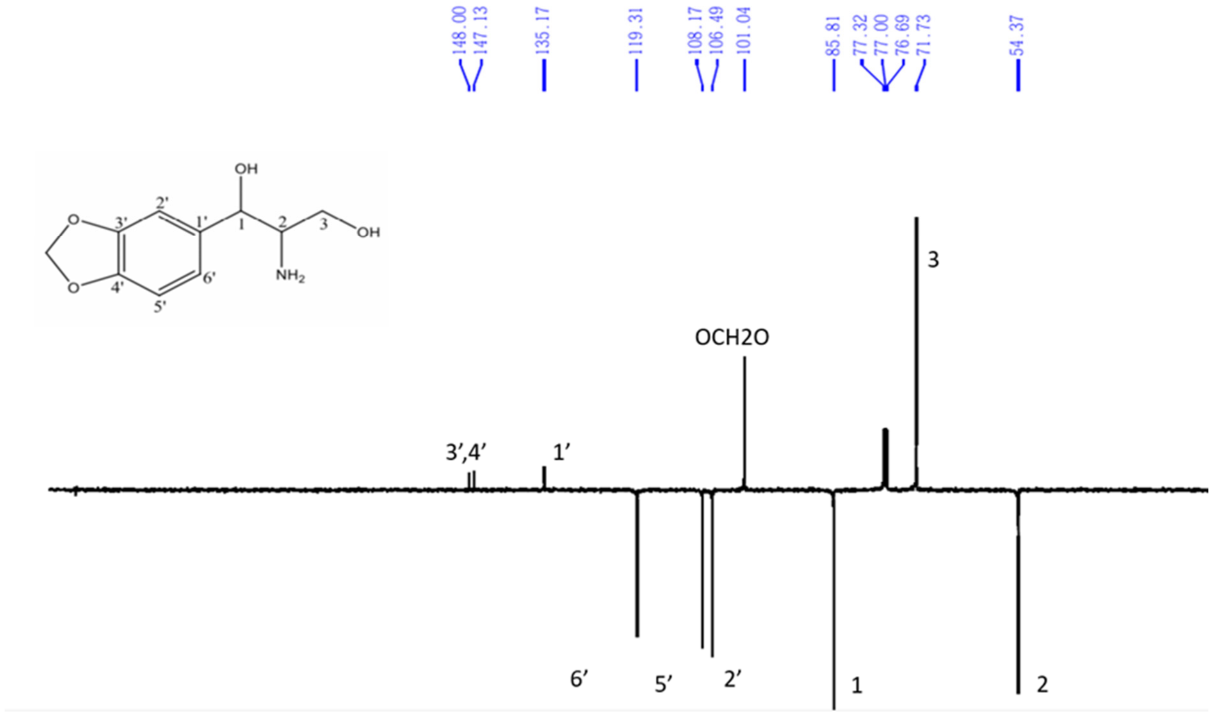

Figure S4. APT spectrum of compound 1

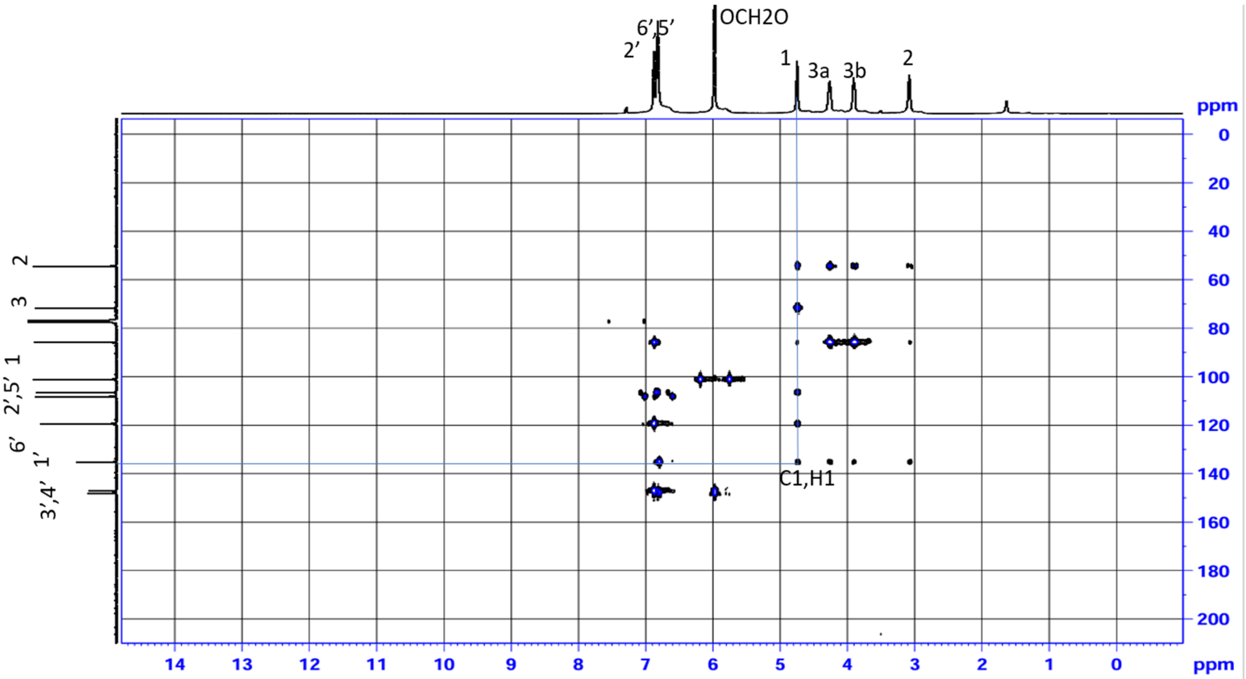

Figure S5. HMBC spectra of compound 1

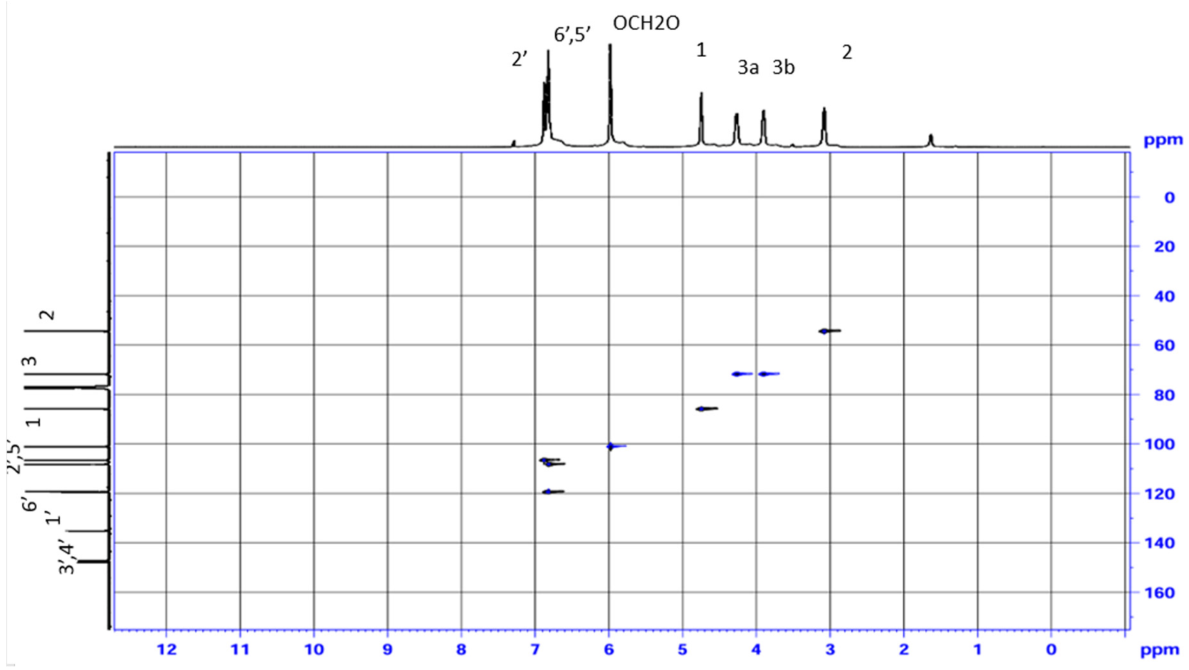

Figure S6. HSQC spectra of compound **1**

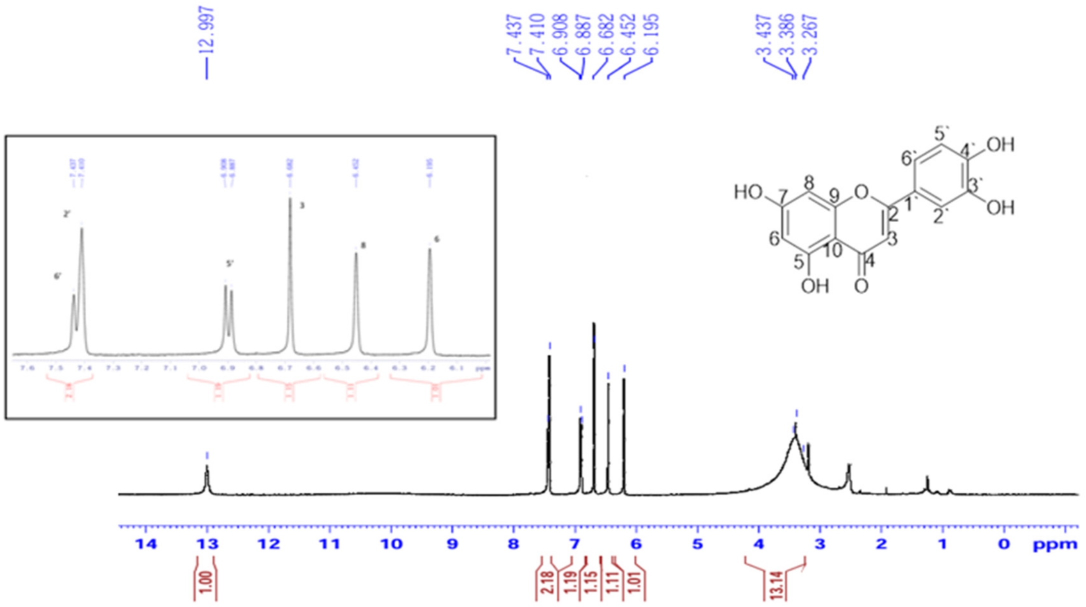

Figure S7. ^{1}H-NMR spectrum of compound 2

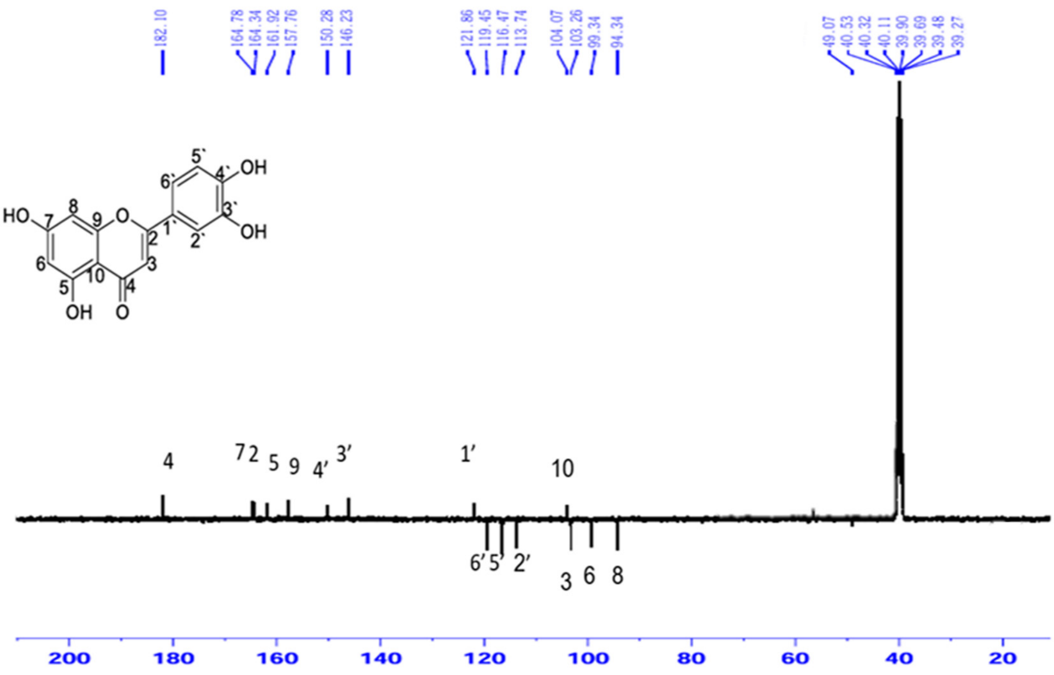

Figure S8. APT spectrum of compound **2**

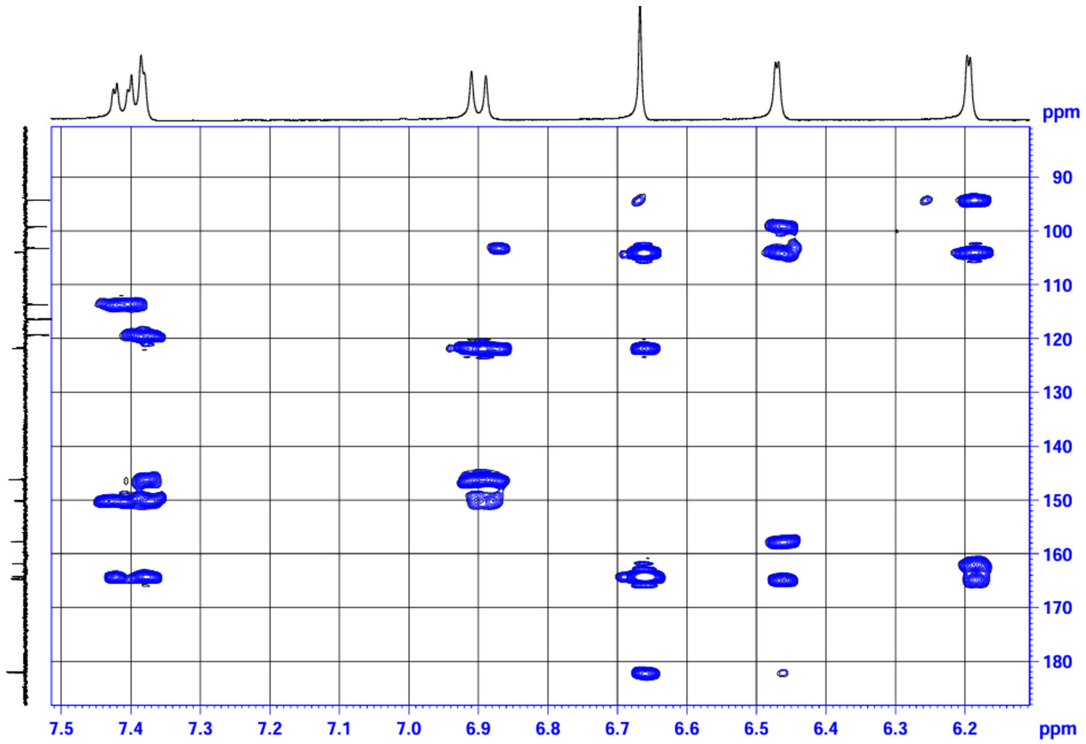

Figure S9. HMBC spectrum of compound **2**

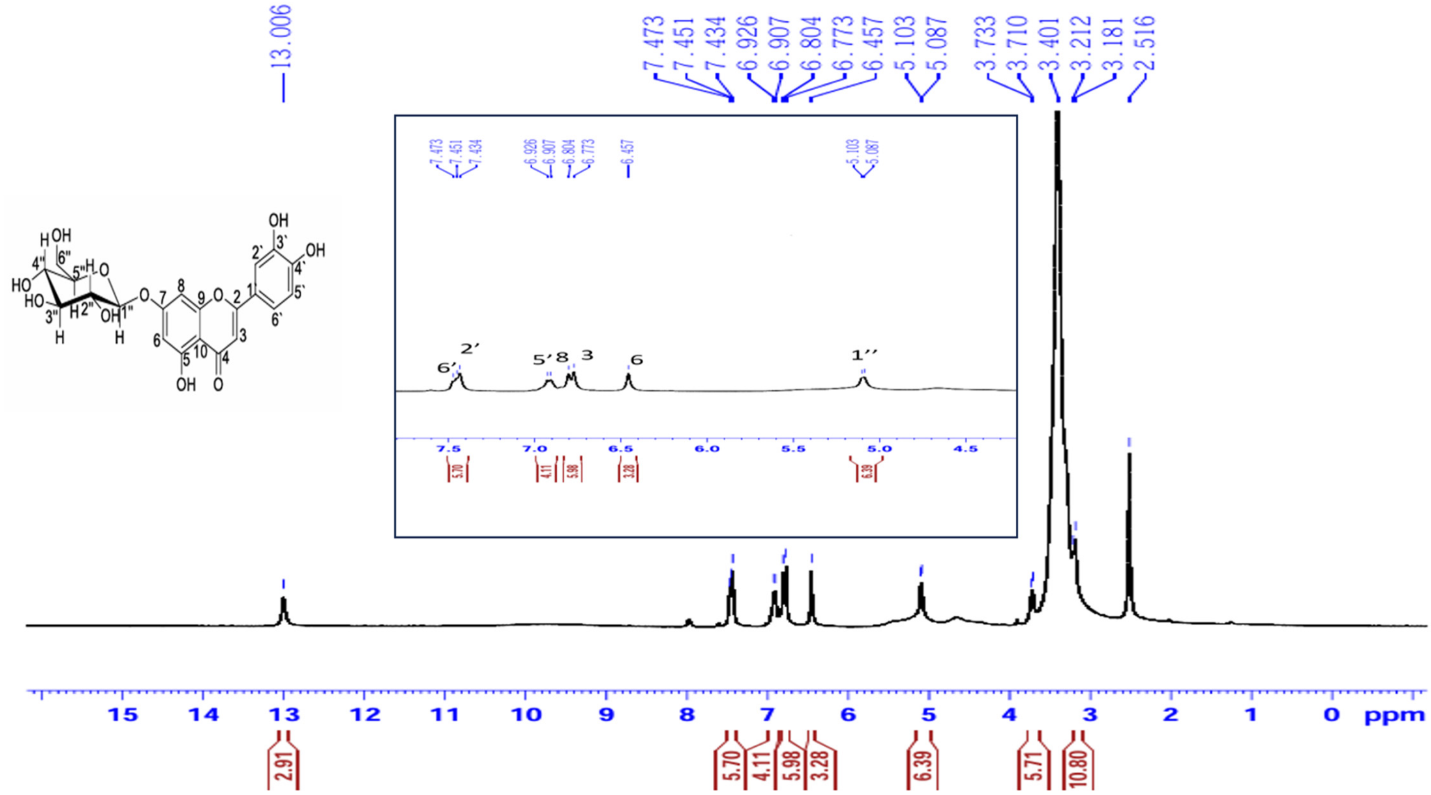

Figure S10. ^{1}H-NMR spectrum of compound **3**

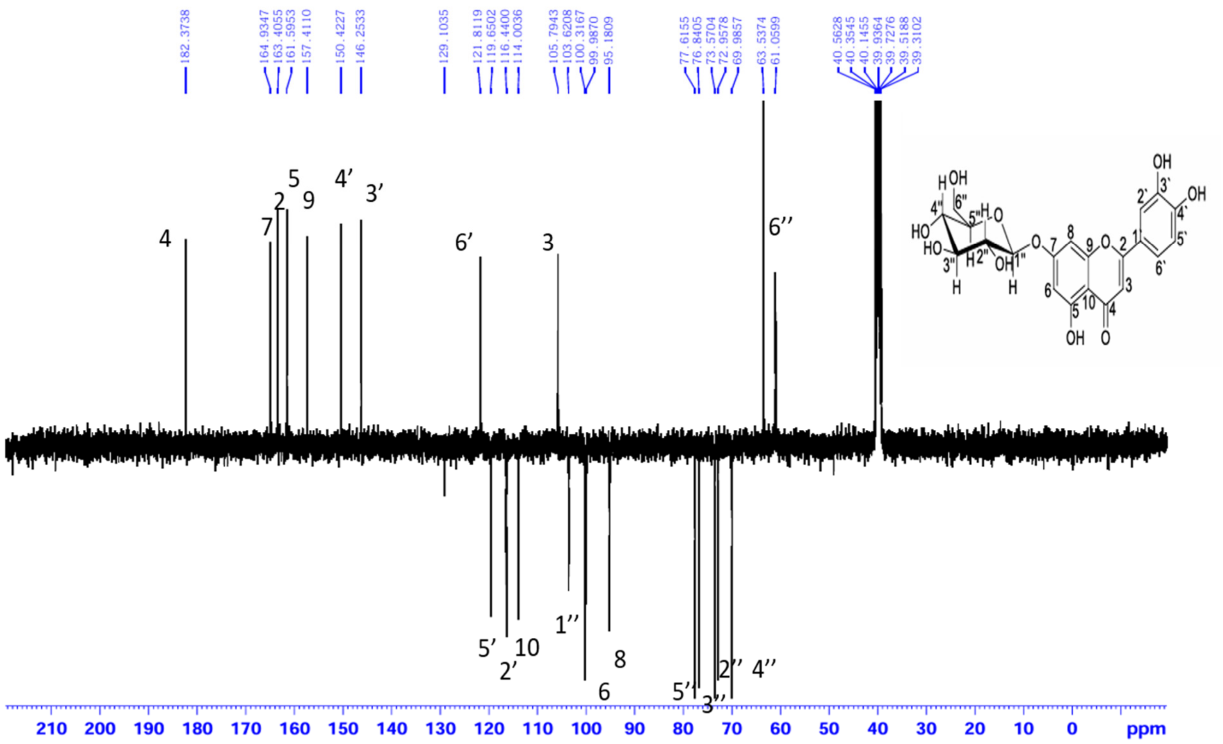

Figure S11. APT spectrum of compound 3

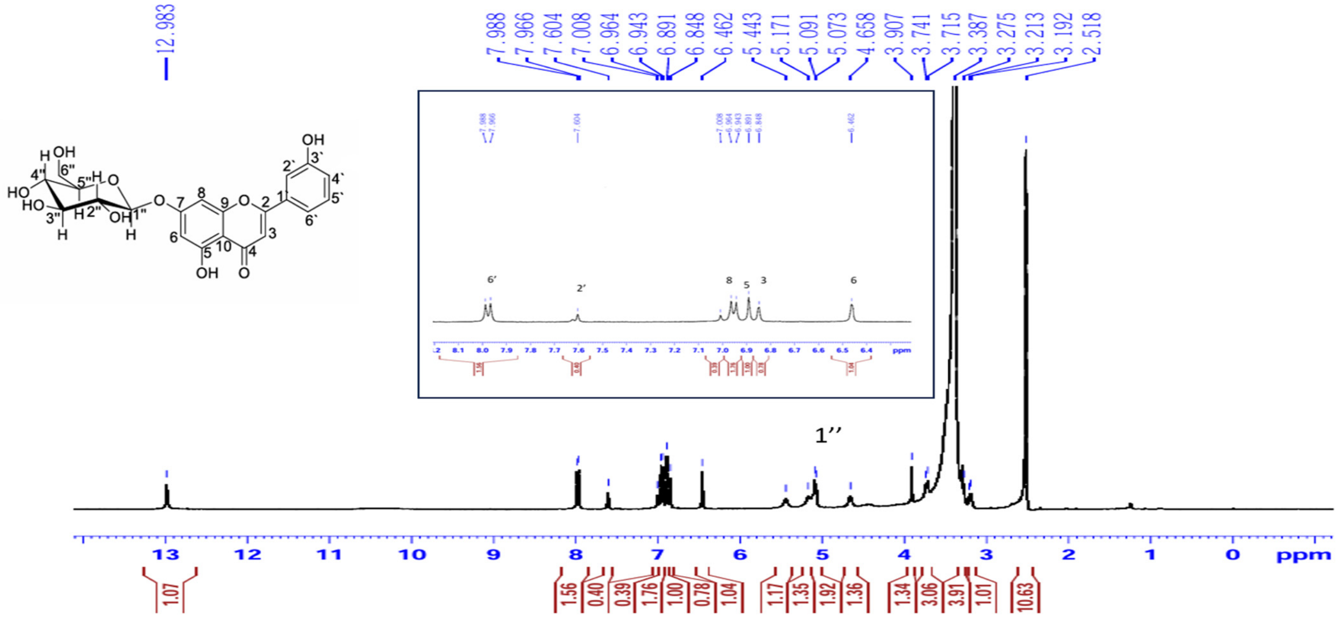

Figure S12. ^{1}H-NMR spectrum of compound 4

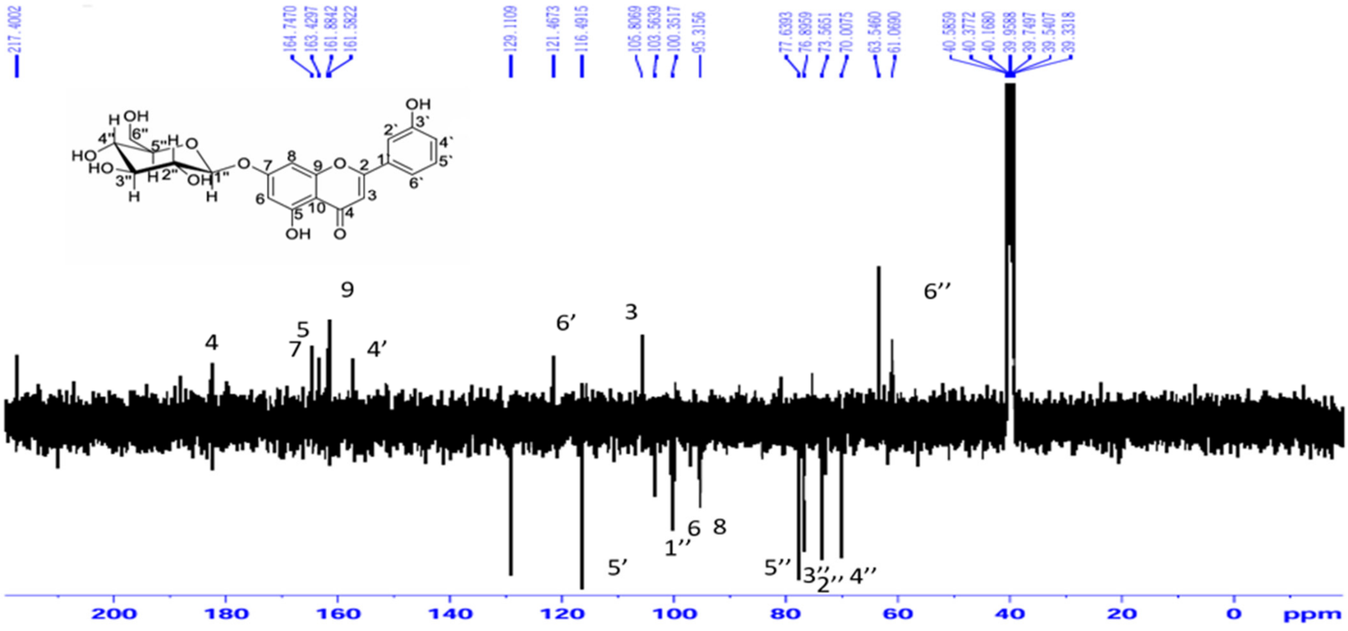

Figure S13. APT spectrum of compound 4

*Chemical shifts (δ) are expressed in ppm and coupling constants (J) in Hz.1H- and 13C-NMR were measured in CD3OD at 400 MHz and 100 MHz, respectively

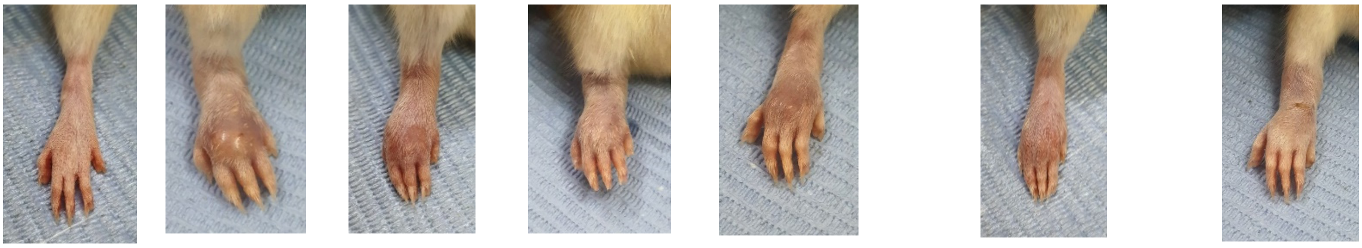

**Normal**	**control**	**Lettuce seed cake extract**	**(Compound (1) 20 mg/kg**	**Compound (1) (40 mg/kg)**	**Combination (Ibuprofen 10 mg/ kg +compound (1) (20mg/kg))**	**Ibuprofen**

Figure S14. treatments effect on carrageenan-induced paw oedema

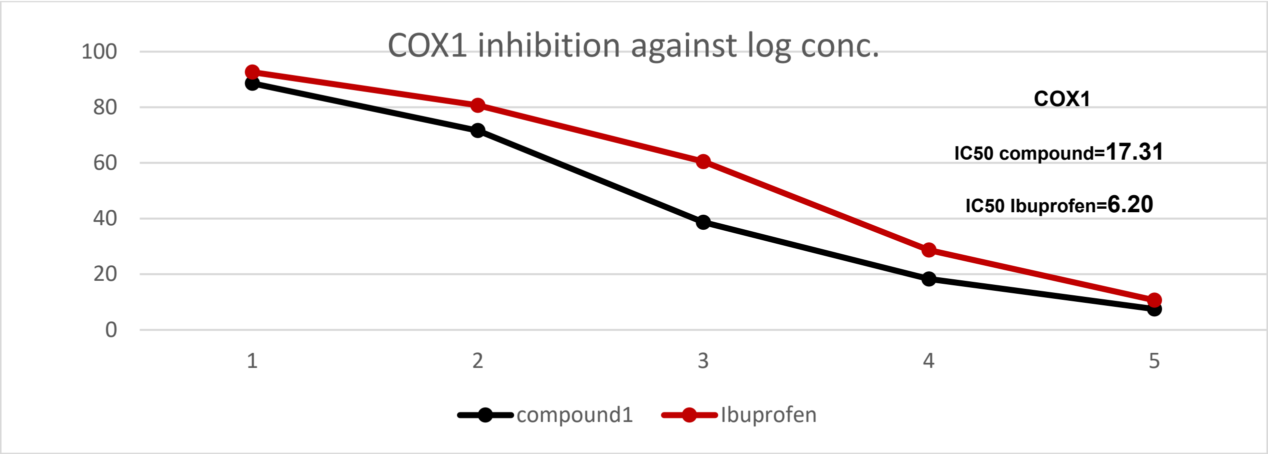

Figure S15. Cox1 inhibition assay for compound 1 against Ibuprofen standard

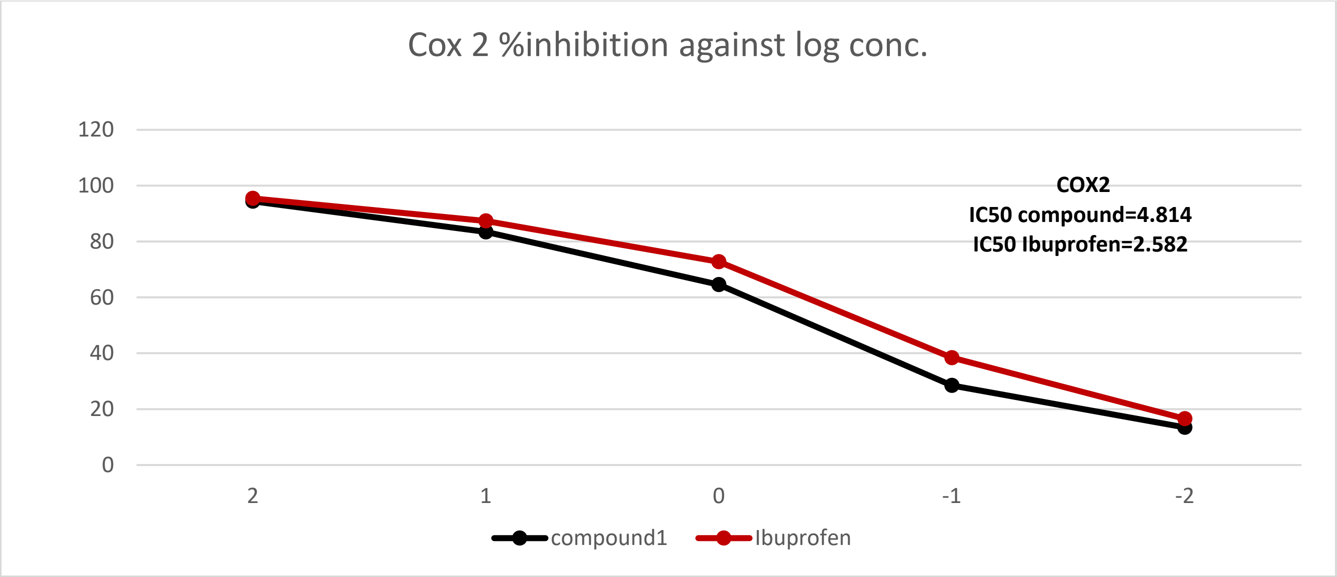

Figure S16. Cox2 inhibition assay for compound *1* against Ibuprofen standard

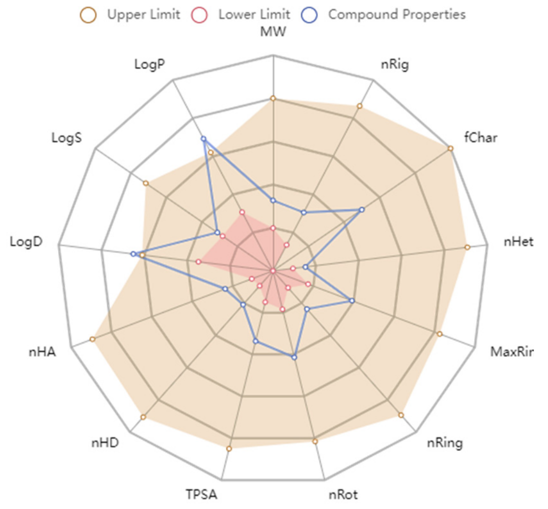

ibuprofen

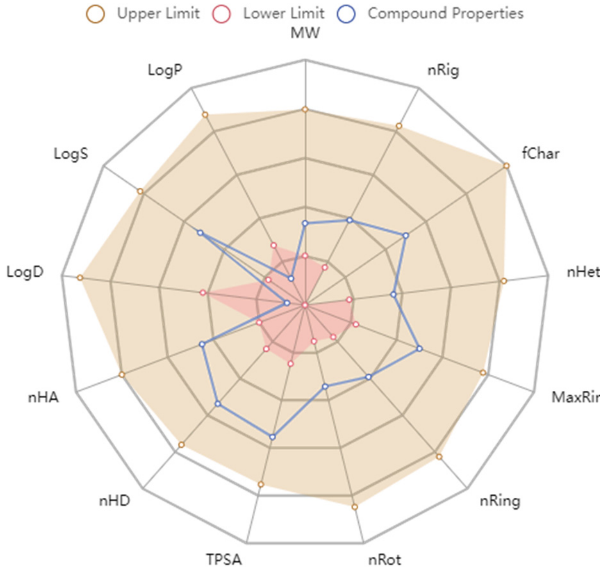

compound 1

Figure S17. ADMET radar Chart for both molecules

Table S1: Antimicrobial activity index of methanolic total extracts of chosen plants seedcakes and different fractions of lettuce seedcake extract (1mg/mL).

	***E. coli* %**		***S. aureus* %**		***C. Albicans* %**	
	**Diameter of inhibition zone (mm)**	**%Activity index**	**Diameter of inhibition zone (mm)**	**%Activity index**	**Diameter of inhibition zone (mm)**	**%Activity index**
**Seed cake total methanolic extract:**						
Lettuce	2.8 ± 0.29	11.86	3.75±0.25	16.4	6.83±0.29	26.4
Black seed	NA*	-----	NA*	-----	NA*	-----
Flaxseed	NA*	-----	1.75±0.25	7.7	4.75±0.25	18.3
Rocket	NA*	----	1.8±0.2	7.9	5.85±0.13	22.6
**Lettuce fractions:**						
Petroleum ether fraction	5.8 ± 0.15	24.4	6.8±0.21	29.7	9.83±0.15	38
Methylene chloride fraction	15.1 ± 0.76	63.26	18.7±0.28	81.6	16.77±0.25	65.4
Ethyl acetate fraction	9.8 ± 0.2	41.1	14.9±0.1	65.2	16.87±0.15	64.7
Ampicillin	23.9 ± 0.15	100	22.87±0.15	100	NA*	----
Clotrimazole	NA*	----	NA*	----	25.9±0.1	100

*NA = No Activity

Table S2: Interaction tested drug with COX1

Interaction	Distance	Category	Type
A:ASN59:HD21 - :UNK1:N	2.46309	Hydrogen Bond	Conventional Hydrogen Bond
:UNK1:H - A:ASN77:OD1	2.832	Hydrogen Bond	Conventional Hydrogen Bond
A:PHE81 - :UNK1	4.73928	Hydrophobic	Pi-Pi T-shaped
A:GLY55:C,O;GLY56:N - :UNK1	3.76329	Hydrophobic	Amide-Pi Stacked
A:PHE81 - :UNK1	4.79014	Hydrophobic	Pi-Alkyl

Table S3: interactions of ibuprofen and COX1

Interaction	Distance	Category	Type
A:PHE81 - :UNL1	4.94701	Hydrophobic	Pi-Pi T-shaped
A:GLY55:C,O;GLY56:N - :UNL1	4.19387	Hydrophobic	Amide-Pi Stacked
:UNL1:C - A:PRO51	4.38441	Hydrophobic	Alkyl
:UNL1:C - A:LEU84	4.60427	Hydrophobic	Alkyl

Table S4: interaction between tested drug and COX2

Interaction	Distance	Category	Type
:UNK1:H - A:GLN195:O	2.2423	Hydrogen Bond	Conventional Hydrogen Bond
:UNK1:H - A:GLN195:O	2.83772	Hydrogen Bond	Conventional Hydrogen Bond
:UNK1:H - A:SER197:OG	1.82539	Hydrogen Bond	Conventional Hydrogen Bond
A:PRO166 - :UNK1	4.93699	Hydrophobic	Alkyl
:UNK1 - A:LEU136	5.31885	Hydrophobic	Alkyl
:UNK1 - A:PRO166	4.27327	Hydrophobic	Pi-Alkyl

Table S5: interaction between ibuprofen and COX2

Interaction	Distance	Category	Type
A:ALA2 - :UNL1	3.96102	Hydrophobic	Alkyl
A:ALA2 - :UNL1:C	4.12993	Hydrophobic	Alkyl
:UNL1 - A:ALA2	4.50205	Hydrophobic	Pi-Alkyl
:UNL1 - A:PRO166	5.13645	Hydrophobic	Pi-Alkyl

Table S6: ADMET result

**compound**	**ibuprofen**	**tested**
LogS	-3.701	-1.61
LogD	3.318	-0.368
LogP	3.687	-0.763

Pgp-inh	0	0.001
Pgp-sub	0.002	0.13
HIA	0.003	0.004
F(20%)	0.002	0.002
F(30%)	0.001	0.002
Caco-2	-4.377	-5.123
MDCK	3.55E-05	2.58E-05
BBB	0.463	0.326
PPB	94.37%	31.56%
VDss	0.238	0.863
Fu	3.65%	72.23%
CYP1A2-inh	0.079	0.846
CYP1A2-sub	0.317	0.145
CYP2C19-inh	0.057	0.035
CYP2C19-sub	0.91	0.677
CYP2C9-inh	0.416	0.008
CYP2C9-sub	0.982	0.171
CYP2D6-inh	0.004	0.802
CYP2D6-sub	0.101	0.583
CYP3A4-inh	0.014	0.469
CYP3A4-sub	0.195	0.248
CL	0.778	5.73
T12	0.687	0.618
hERG	0.018	0.07
H-HT	0.431	0.166
DILI	0.946	0.047
Ames	0.004	0.054
ROA	0.538	0.079
FDAMDD	0.015	0.029
SkinSen	0.17	0.129
Carcinogenicity	0.072	0.368
EC	0.018	0.011
EI	0.586	0.075
Respiratory	0.066	0.556
BCF	0.462	0.381
IGC50	3.418	2.415
LC50	3.509	2.623
LC50DM	3.938	3.564
NR-AR	0.044	0.488
NR-AR-LBD	0.003	0.208
NR-AhR	0.008	0.443
NR-Aromatase	0.009	0.016
NR-ER	0.145	0.113
NR-ER-LBD	0.018	0.007

NR-PPAR-gamma	0.795	0.003
SR-ARE	0.029	0.047
SR-ATAD5	0.004	0.012
SR-HSE	0.044	0.017
SR-MMP	0.013	0.028
SR-p53	0.006	0.028
MW	206.13	211.08
Vol	231.882	202.652
Dense	0.889	1.042
nHA	2	5
nHD	1	4
TPSA	37.3	84.94
nRot	4	3
nRing	1	2
MaxRing	6	9
nHet	2	5
fChar	0	0
nRig	7	10
Flex	0.571	0.3
nStereo	1	2
NonGenotoxic_Carcinogenicity	0	0
LD50_oral	0	0
Genotoxic_Carcinogenicity_Mutagenicity	0	0
SureChEMBL	0	0
NonBiodegradable	0	0
Skin_Sensitization	0	1
Acute_Aquatic_Toxicity	0	0
Toxicophores	0	1
QED	0.822	0.643
Synth	2.192	3.05
Fsp3	0.462	0.4
MCE-18	16	34.571
Natural Product-likeness	0.137	0.87
Alarm_NMR	0	0
BMS	0	0
Chelating	0	0
PAINS	0	0
Lipinski	Accepted	Accepted
Pfizer	Rejected	Accepted
GSK	Accepted	Accepted
GoldenTriangle	Accepted	Accepted
